# TOLOMEO, a Novel Machine Learning Algorithm to Measure Information and Order in Correlated Networks and Predict Their State

**DOI:** 10.3390/e23091138

**Published:** 2021-08-31

**Authors:** Mattia Miotto, Lorenzo Monacelli

**Affiliations:** 1 Department of Physics, Sapienza University of Rome, 00184 Rome, Italy; 2 Center for Life Nano- & Neuro Science, Istituto Italiano di Tecnologia, 00161 Rome, Italy

**Keywords:** entropy, maximum entropy, hopfield model, machine learning

## Abstract

We present ToloMEo (TOpoLogical netwOrk Maximum Entropy Optimization), a program implemented in C and Python that exploits a maximum entropy algorithm to evaluate network topological information. ToloMEo can study any system defined on a connected network where nodes can assume N discrete values by approximating the system probability distribution with a Pottz Hamiltonian on a graph. The software computes entropy through a thermodynamic integration from the mean-field solution to the final distribution. The nature of the algorithm guarantees that the evaluated entropy is variational (i.e., it always provides an upper bound to the exact entropy). The program also performs machine learning, inferring the system’s behavior providing the probability of unknown states of the network. These features make our method very general and applicable to a broad class of problems. Here, we focus on three different cases of study: (i) an agent-based model of a minimal ecosystem defined on a square lattice, where we show how topological entropy captures a crossover between hunting behaviors; (ii) an example of image processing, where starting from discretized pictures of cell populations we extract information about the ordering and interactions between cell types and reconstruct the most likely positions of cells when data are missing; and (iii) an application to recurrent neural networks, in which we measure the information stored in different realizations of the Hopfield model, extending our method to describe dynamical out-of-equilibrium processes.

## 1. Introduction

Predictability is often the ultimate goal that drives the study of various physical phenomena [1]. For example, when we investigate the dynamics of a falling body or the interactions between two molecules, we understand the phenomena when we are able to foretell the body’s trajectory or the effect of a novel medical drug. Our capability of making predictions is linked with the quantity of information we gather about the system we are considering. In this respect, the connection between entropy and information is regarded as a milestone of information theory [2]. In a very general way, entropy quantifies our knowledge of the probability of the system to assume its states [3,4]. Consequently, knowing the entropy allows us to set the limit to the information that we can extract from observations and, more generally, to the predictability of the system.

The concept of entropy was first introduced in thermodynamics, but its application ranges in many fields, such as physics [5], economics [4,6], or biology [3,7,8,9,10,11]. Indeed, the general formulation of statistical mechanics and information theory opened the way for the understanding of many features of complex systems. Among many possible examples, entropy has been employed even in economics, where the awareness of markets entropy allows one to maximize the investment profits [4]. Despite its broad applicability, measuring the entropy of a complex system has proven to be quite challenging [8]. In fact, the Shannon definition of entropy relies on the system probability function P, whose degrees of freedom grow exponentially as a function of the number of possible states the system can access, often making its computation unfeasible. The maximum entropy (MaxEnt) approach helped to solve this problem [3]. In fact, given a constrain on a set of observables $\left\{x_{i}\right\}$, MaxEnt finds the less biased probability function for the system that describes the observables, i.e., the probability distribution that maximizes entropy among all possible distributions that satisfy the constraints on the chosen observables. Thanks to the variational principle, the entropy associated with the MaxEnt distribution is always an upper bound to the exact entropy. Furthermore, the arbitrariness of the observables set makes it possible to control the accuracy of the approximation: increasing the number of constraints, we reduce the space accessible to the possible probability distributions.

Over the past decade, many works employed MaxEnt to analyze different biological problems, ranging from the study of neural populations to the determination of macromolecular structures, and the inference of regulatory networks [12,13,14,15,16,17]. From the identification of DNA specific binding sites [18] to the comprehension of collective behavior in large animal groups [19,20], and to the modeling of ecological systems [21,22].

Given the vast applicability of the maximum entropy principle, here, we present ToloMEo (TOpoLogical netwOrk Maximum Entropy Optimization), a program able to solve the MaxEnt algorithm for generic networks whose nodes can assume a certain, finite, number of states. In practice, ToloMEo finds the less-biased probability distribution that constrains the average density of states of the network nodes and the number of near-neighbor couples for each possible set of states. Once trained, ToloMEo is able to reconstruct missing data from a source like a machine learning approach. In addition, the strong inference power of the maximum entropy principle allows one to train the system on a limited set of data, which can also be a small portion of the source we want to study. The method is very general and applicable to any network topology. Examples are linear chains, 2D lattices (such as images), multidimensional lattices, or complex networks.

We first present the theoretical background and describe the algorithm; then, we discuss three different cases of study: (i) we investigate a model of a minimal ecosystem composed of two interacting species in a 2D lattice; (ii) we apply ToloMEo, for the first time, to measure order and information stored in biological images of cell populations, where different kinds of cells are colored with distinct fluorescence markers. Finally, (iii) we analyze the dynamics of the Hopfield model, a recurrent neural network (RNN) whose connectivity matrix may present different degrees of asymmetry and dilution [23,24]. ToloMEo is released as a web server app freely accessible at http://circe.iit.uniroma1.it:9205 (25 August 2021).

## 2. Method Overview

**Figure 1:**
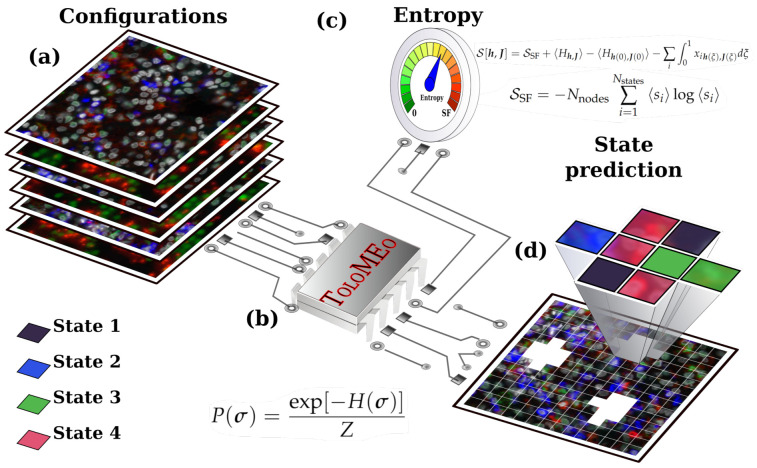
Sketch of the ToloMEo protocol. (**a**) ToloMEo requires in input a set of configurations of the real system. The input data must be provided as a network with nodes that can assume a discrete number of states and a connectivity matrix specifying the topological connections between the nodes. In the sketch, configurations are 2D images of cell populations, with cells colored with different fluorescence dyes. The image is divided into a 2D uniform grid. Each point in the grid is a node; the color of the image in each node represents the state, while the connectivity matrix is a near-neighbor 2D lattice. (**b**) ToloMEo takes as input the set of configurations and returns the maximum entropy probability distribution, *P*, that better reproduces a set of chosen observables. (**c**) Starting from the optimiization path, the MaxEnt entropy, *S*, can be evaluated using Equation (12). (**d**) The obtained probability distribution can be used to generate novel configurations and to predict the spatial disposition of some states, keeping fixed the others.

ToloMEo is an inferential protocol capable of learning the probability distribution that maximizes the entropy of a system composed (i) by a set of nodes, assuming discrete states, and (ii) defined on a symmetric (if node *A* is linked to *B*, then *B* is linked to *A*), not-weighted network. In particular, ToloMEo looks for the Hamiltonian that reproduces a certain set of observables while maximizing the system’s entropy. Indeed, to encode the system network topology in the Hamiltonian, ToloMEo constrains mean-field observables (average number of nodes in each possible state) and near-neighbor pairwise correlations (between all possible couples of states). The resulting Hamiltonian, that maximizes entropy, is a Potts Hamiltonian on the same network [21]. The entropy maximization ensures that the solution found by ToloMEo is the least-biased solution that satisfies the imposed constraints on the average observables [25]. Thus, it confers robustness to the method with respect to over-fitting. Figure 1 provides a schematic representation of the ToloMEo protocol.

The training procedure, which determines the parameter of the Hamiltonian that best reproduces the average values of the constraints, is the pivotal part of the algorithm and works in the following way: (i) we start from a non-interacting Hamiltonian that reproduces only the average number of nodes of the network in each state. (ii) We perform a Monte Carlo calculation and find the direction for the parameters of the trial interacting Hamiltonian (including pairwise interaction between neighbor sites) to improve the agreement with the average observables in the training set. (iii) We update the interacting Hamiltonian and iterate from step (ii) until convergence (the ToloMEo’s observables and those in the training set are compatible within the stochastic accuracy). In Section 3, we provide the details of how the algorithm is implemented. At the end of the training, ToloMEo provides the entropy of the system without any extra computation. The entropy is a score of the method and a measurement of the predictability of the system (if low, predictions are accurate; if high, predictions have high uncertainty). The obtained entropy is always an upper bound to the exact entropy of the process that generated the training set. Thus, it provides an essential insight into the studied process.

ToloMEo is computationally heavier to train than other machine learning approaches (such as neural networks). In fact, to evaluate the cost function, we need to run a Metropolis algorithm. However, thanks to the much smaller number of parameters on which it depends with respect to neural networks, the cost function usually has only one well-defined minimum, making the parameter optimization straightforward. Besides, the final converged result carries physical insight into the process under study, as it provides effective interaction between states in the system. Notably, this insight proved to be enough to infer protein residue contacts within sets of homologous proteins [15].

Moreover, differently from commonly used machine learning approaches, ToloMEo does not learn specific patterns in the training data set, but, given a set of observables, it constructs the least-biased probability distribution that reproduces an ensemble with the same average values of the chosen observable set as those in the training set. Thus, the outcome of ToloMEo is not the prediction of a feature learned from the training data, but an effective Hamiltonian that allows one to extract configurations of the systems with the same probability as the original process that generated the training data. Therefore, ToloMEo can predict the status of the network when some information is missing. A typical example is a case when we have a network whose nodes can assume several states, and our data cannot distinguish between two or more states. ToloMEo solves the problem by simulating the missing states, finding the most probable solution.

ToloMEo is robust against over-fitting and typical learning patterns of the training set and requires a small training set and no test set, contrarily to many other machine learning approaches. In particular, of all the information in the training set, ToloMEo only extracts the average number of states in the whole network and the near-neighbor’s correlations between all possible states. By construction, ToloMEo correctly reproduces all these features, within the stochastic error easily measurable directly from the training set. Moreover, since ToloMEo never sees the actual configurations of the training set (but only the average value of the chosen observables), it is possible to reuse the same training set to test the method’s validity on different observables not employed in the training procedure.

## 3. Materials and Methods

ToloMEo takes in input an ensemble of configurations of the system we want to analyze. The system must be defined as a network with a certain number of nodes ($N_{\mathrm{nodes}}$), each assuming one of $N_{\mathrm{states}}$ possible states, and a static connectivity matrix, defining the topological connections between couples of nodes. Such configurations constitute the training set. In the following, we refer to σ as a specific network configuration, and with $\sigma_{k}$ to the status of the *k*-th node of σ configuration. The discrete states, each node can assume, are represented by integer numbers between 0 and $N_{\mathrm{states}}-1$.

ToloMEo trains a specific model to reproduce the probability distribution of finding a configuration σ produced by the same source that generated the training set. This probability distribution P(σ) can be represented by an auxiliary Hamiltonian, H(σ) defined as

(1) $$P\left(\sigma \right)=\frac{\exp\left[-H(\sigma )\right]}{Z},\;\mathrm{with}\;Z=\sum_{\sigma }\exp\left[-H(\sigma )\right]\;.$$

For each configuration of the system, we evaluate two different kinds of observables. The density of states *i* on a configuration σ of the network, defined as

(2) $$s_{i}\left(\sigma \right)=\frac{1}{N_{\mathrm{nodes}}}\sum_{k=1}^{N_{\mathrm{nodes}}}\delta_{\sigma_{k},i},$$

and the density of near neighbor couples between state *i* and *j*,

(3) $$c_{ij}\left(\sigma \right)=\frac{1}{N_{\mathrm{links}}}\sum_{\begin{array}{c} k,h=1\\ h,k\;\mathrm{near}\;\mathrm{neighbors} \end{array}}^{N_{\mathrm{nodes}}}\delta_{\sigma_{h},i}\delta_{\sigma_{k},j}\;.$$

Here, δ indicates the Kronecker delta, $N_{\mathrm{links}}$ corresponds to the total number of couples of connected nodes, and the sum in the $c_{ij}$ expression is performed only on couples of nodes that are connected by a link in the network (near neighbors). The average values of these observables on the whole training set are given by:

(4) $$\langle s_{i}\rangle =\frac{1}{N_{\mathrm{train}}}\sum_{\sigma_{k}\in \mathrm{training}\;\mathrm{set}}s_{i}\left(\sigma_{k}\right)\;,\;\mathrm{and}\;\langle c_{ij}\rangle =\frac{1}{N_{\mathrm{train}}}\sum_{\sigma_{k}\in \mathrm{training}\;\mathrm{set}}c_{ij}\left(\sigma_{k}\right)\;,$$

where $N_{\mathrm{train}}$ is the number of configurations in the training set.

To simplify the notation, we introduce a vector x to describe the status of the system, which is given by the average value of the target observables over an ensemble:

(5) $$x=\left(\begin{array}{cccccc} \langle s_{1}\rangle & \cdots & \langle s_{N_{\mathrm{states}}}\rangle & \langle c_{11}\rangle & \cdots & \langle c_{N_{\mathrm{states}},N_{\mathrm{states}}}\rangle \end{array}\right)\;.$$

At present, ToloMEo restricts to distributions that only reproduce $\langle s_{i}\rangle$ and $\langle c_{ij}\rangle$ correctly in the training set. Therefore, the training set is used only to extract the average number of states in each configuration and the near neighbor’s correlations between states. Among all the possible probability distributions P(σ) that satisfy these constraints, ToloMEo chooses the one that maximizes the entropy, providing the least-biased solution [25]. It is possible to prove [26] that this probability distribution is obtained with an auxiliary Hamiltonian H(σ) with near-neighbor interaction (a Potts Hamiltonian, i.e., a multi-state Ising model) of the form

(6) $$H_{h,J}\left(\sigma \right)=\sum_{i=1}^{N_{\mathrm{states}}}h_{i}s_{i}\left(\sigma \right)+\sum_{i,j=1}^{N_{\mathrm{states}}}J_{ij}c_{ij}\left(\sigma \right)\;.$$

The Hamiltonian depends on the vector h and the symmetric matrix J. Those are the parameters that ToloMEo trains to enforce $\langle s_{i}\rangle$ and $\langle c_{ij}\rangle$ to reproduce the values obtained from the training set.

We indicate with $x_{h,J}$ the expression in Equation (5) when the averages are computed with the ensemble extracted from the Hamiltonian defined by h and J. Conversely, dropping the indices, we refer to averages taken on the training set, i.e., the vector of features that the trained Hamiltonian must reproduce.

It is important to note that not all the values of $h_{i}$ and $J_{ij}$ are independent, as the Hamiltonian has gauge freedom. Indeed, the observables have linear dependencies between themselves. For example, the sum of all possible states in the network is equal to the total number of nodes, which in terms of densities translates in:

(7) $$\sum_{i=1}^{N_{\mathrm{states}}}s_{i}\left(\sigma \right)=1\;.$$

From Equation (7), we obtain an arbitrary gauge choice on the $h_{i}$ values: all $h_{i}$ values shifted by a constant Δ produce the same probability distribution:

(8) H(h+Δ,J)=Δ+H(h,J).

Analogous relations hold for the $\langle c_{ij}\rangle$ coefficients and between $\langle s_{i}\rangle$ and $\langle c_{ij}\rangle$.

In order to fix the gauge, we compute the covariance matrix Σ of the target observables on the training set. Then, we diagonalize Σ, and project out the subspace defined by the kernel of Σ. We described this procedure in more detail in Ref. [21].

The optimization of the parameters proceeds by successive Monte Carlo–Metropolis simulations: for a fixed choice of h and J, ToloMEo runs a Metropolis simulation and extracts an ensemble of equilibrium configurations. This ensemble is used to compute the average of the constrained observables over the auxiliary Hamiltonian ($x_{h,J}$). Next, we define a $\chi^{2}$ variable (note that we project out the kernel of Σ from ($x-x_{h,J}$) as

(9) $$\chi^{2}=\left(x-x_{h,J}\right)\Sigma^{-1}\left(x-x_{h,J}\right)\;.$$

The values of h and J are optimized with a conjugate gradient algorithm to minimize $\chi^{2}$. The explicit expression of the gradients of Equation (9) is reported and derived in Ref. [21].

To avoid performing a new Metropolis–Monte Carlo at each step of the optimization, we employ an importance sampling technique that consists of reusing the ensemble generated by a certain Hamiltonian assigning a weight for each configurations equal to

(10) $$\rho_{i}=\frac{\exp\left[-H_{h,J}\left(\sigma_{i}\right)+H_{h_{0},J_{0}}\left(\sigma_{i}\right)\right]}{\sum_{j}\exp\left[-H_{h,J}\left(\sigma_{j}\right)+H_{h_{0},J_{0}}\left(\sigma_{j}\right)\right]}\;.$$

where $h_{0},J_{0}$ are the values on which we run the last Monte Carlo–Metropolis simulation.

Unlike the procedure introduced in Ref. [21], here, we employ a more robust criterion to check whether the ensemble still provides reliable averages. We measure the effective sample size ratio and check if it is above a user-defined threshold η (usually about 0.5):

(11) $$\frac{N_{\mathrm{conf}}\sum_{i}\rho_{i}^{2}}{{\left(\sum_{i}\rho_{i}\right)}^{2}}>\eta \;,$$

where $N_{\mathrm{conf}}$ is the number of configurations in the extracted ensemble. If the inequality (11) is not satisfied, a new Monte Carlo–Metropolis algorithm is performed with the last h,J values and the ensemble is updated. The use of Equation (11) to evaluate the importance sampling proved to be very efficient in similar algorithms [27,28].

**Figure 2:**
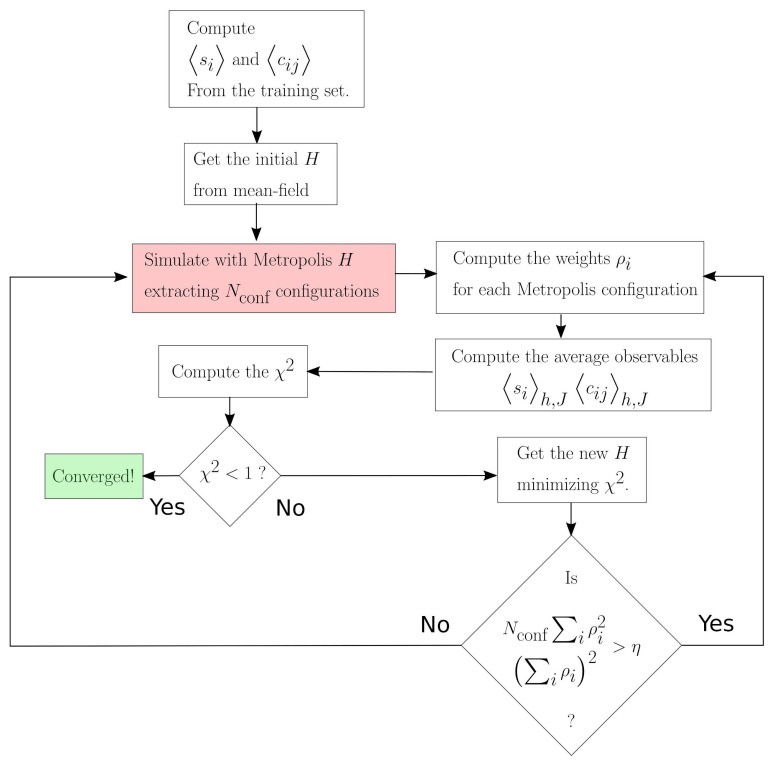
Flowchart of the ToloMEo algorithm. The red cell, namely the Metropolis simulation of Hamiltonian, *H*, is the most computationally expensive part of the calculation, while, in comparison, all the other procedures are almost instantaneous. For this reason, the overall computational cost of the algorithm depends only on the number of times the flowchart passes through that cell.

The program converges when the $\chi^{2}$ divided by the number of degrees of freedom is lower than a user-given threshold (below 1). The complete flowchart of the ToloMEo algorithm is reported in Figure 2.

During the minimization of the $\chi^{2}$ (Equation (9)), ToloMEo saves the full path of the Hamiltonian parameters h,J and the corresponding average observables, $x_{h,J}$. Thus, without any additional computational effort, we can compute the entropy of the probability distribution as

(12) $$S\left[h,J\right]=S_{\mathrm{SF}}+\langle H_{h,J}\rangle -\langle H_{h(0),J(0)}\rangle -\sum_{i}\int_{0}^{1}{x_{i}}_{h(\xi ),J(\xi )}d\xi \;,$$

where ξ is a variable that parametrizes the evolution of the Hamiltonian from the starting guess h(ξ=0),J(ξ=0) to the converged values h(ξ=1),J(ξ=1). The first term of the entropy corresponds to the Shannon–Fano entropy for non-interacting states:

(13) $$S_{\mathrm{SF}}=-N_{\mathrm{nodes}}\sum_{i=1}^{N_{\mathrm{states}}}\langle s_{i}\rangle \log\langle s_{i}\rangle \;.$$

This equation holds as long as the starting condition is the non-interacting solution, where

$$h_{i}\left(0\right)=-\ln\left(\langle s_{i}\rangle \right)\;J\left(0\right)=0\;.$$

Equation (12) is obtained from thermodynamic integration along the training path, and it was derived in Ref. [21].

### Dynamical Maximum Entropy

The procedure applied so far describes time-independent processes: it models the probability of being in a state that does not depend on past conditions.

It is easy to extend the maximum entropy principle to deal with time sequences of configurations ${\left\{\sigma \right\}}_{t}$. In fact, all the properties of the dynamical system are encoded in the probability $P({\left\{\sigma \right\}}_{t})$ of finding any particular time sequence of states ${\left\{\sigma \right\}}_{t}$.

As we did for the static maximum entropy, we can define an auxiliary function H to determine the probability distribution

(14) $$P\left({\left\{\sigma \right\}}_{t}\right)=\frac{\exp\left[-H({\left\{\sigma \right\}}_{t})\right]}{Z}\;.$$

We can then repeat both the theoretical and the computational procedure to model $H({\left\{\sigma \right\}}_{t})$ as we did for the static case, just replacing σ with ${\left\{\sigma \right\}}_{t}$. This choice increases the variety of the constrained observables in the training set (which is composed of time-sequences of states). By choosing only time-independent observables, i.e., observables that do not couple configurations of different timesteps, we obtain the same final result as the static maximum entropy (the observables are averaged in time). On the opposite side, if we introduce observables that depend on time, we obtain a new dynamical representation of the system. ToloMEo, as it is implemented right now, allows one to constrain self-time correlations: i.e., the probability of changing the state of a system in two subsequent timesteps. A similar approach is presented in Ref. [29]. As for the static maximum entropy, also in the dynamical case, ToloMEo can variationally compute the dynamical entropy, defined as:

(15) $$S_{D}=-\sum_{{\left\{\sigma \right\}}_{t}}P\left({\left\{\sigma \right\}}_{t}\right)\ln P\left({\left\{\sigma \right\}}_{t}\right)\;.$$

One of the important features of ToloMEo is the ability to train the probability distribution from a very limited training set, which allows one to train a full dynamical probability distribution of time sequences even from a single time sequence. We show the performances of ToloMEo in dynamical maximum entropy, computing the dynamical entropy of the Hopfield model in Section 4.3.

## 4. Results and Discussion

We present three different case studies that highlight the broad applicability of ToloMEo.

### 4.1. Agent Based Model on 2D Lattice: The EcoLat Model

As the first example of possible application, we discuss the case of an agent-based model defined on a 2D lattice; we note that the generalization to the 3D lattice is straightforward. Agent-based models consist of (i) a set of individuals (the agents) which can assume a determined number of possible states, (ii) a set of rules that dictates the activity of each agent and the interactions with other agents, (iii) a network that identifies which agent interacts to each other. Notably, one can represent many important complex systems on a lattice conserving their essential features [30].

Here, we considered the EcoLat model [21,26] where a minimal ecosystem composed of two species is defined on a 2D lattice. Each site can assume three possible states (i.e., 0, 1, or 2) representing the environment, a prey (fish), or a predator (shark), respectively. A set of rules governs the dynamics of each agent, which can move, breed, or die according to a certain probability (see Ref. [21] for more details).

**Figure 3:**
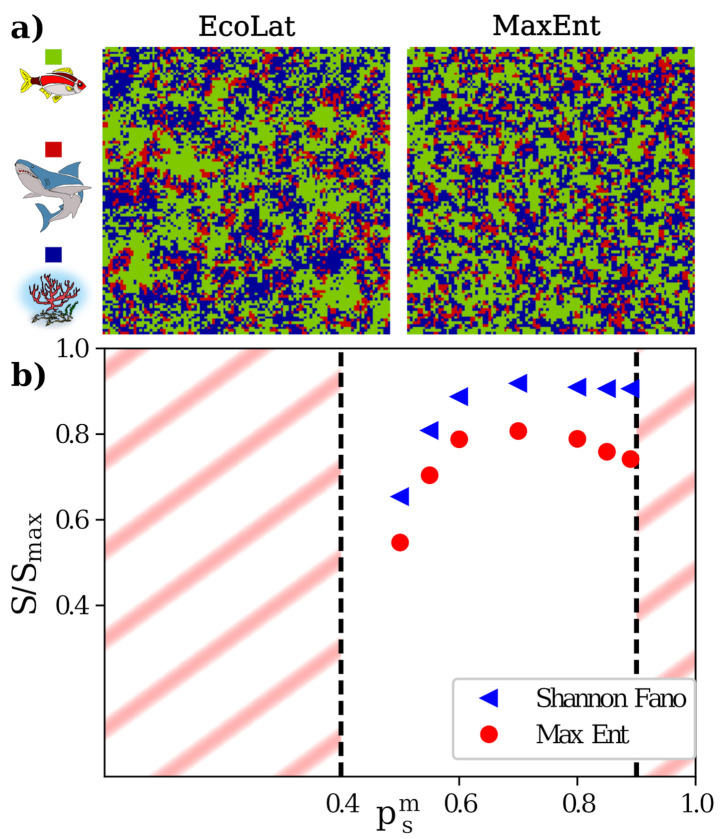
Measuring entropy in a minimal model ecosystem. (**a**) Comparison between the *in silico* ecosystem (EcoLat) and the maximum entropy (MaxEnt) result. On the left, representation of an EcoLat snapshot in the steady-state regime. Fish are colored in green, sharks in red, while blue represents the environment. On the right, we report a configuration extracted from the MaxEnt probability distribution constraining the numbers of prey, predators, and near-neighbor couples. Both simulations ran on a lattice of edge, L=110. (**b**) Entropy per site as a function of predator mobility parameter. Blue triangles indicate the Shannon–Fano entropy, while red circles represent the MaxEnt entropy. Obliques lines underline the ranges of the parameter that lead species to extinction. A difference in behavior of the two entropies manifests in the region $p_{s}^{m}\in \left(0.7,0.9\right)$. These differences outline that structural ordering occurs in the system.

Depending on the choice of the parameters (i.e., the set of probabilities), the system evolves toward either an absorbing state (fish saturation or complete life extinction) or toward a Non-Equilibrium Steady-State (NESS), in which fish and shark densities fluctuate around a constant value. A snapshot of an ‘EcoLat’ NESS configuration is shown in Figure 3a.

In this framework, ToloMEo can be easily applied by selecting a three-state setup with the *‘lattice’* topology and passing in input a set of NESS configurations. As one can see from the ‘MaxEnt’ snapshot in Figure 3a, the general aspect of the system is well reproduced using near-neighbor MaxEnt. Moreover, it is possible to study the behavior of the configurational entropy as a function of the species’ relevant phenotypes. For example, Figure 3b displays the entropy per site of the system normalized by its maximal value (ln3) as a function of the predator mobility. Blue triangles represent the mean-field Shannon–Fano entropy, while red circles show the entropy obtained via the MaxEnt approximation considering near-neighbor correlations. The MaxEnt entropy estimation is always lower than the mean-field result, as expected due to the variational nature of the least entropy principle. We can see a qualitative difference between Shannon–Fano and MaxEnt entropy trends. MaxEnt entropy displays a maximum around $p_{s}^{m}=0.7$, while Shannon–Fano entropy reaches a plateau. An increased difference between Shannon–Fano and MaxEnt entropy is a clear sign that structural ordering occurs, and that MaxEnt entropy effectively considers spatial correlations even beyond near-neighbor ones (see Ref. [21] for more detailed discussions).

### 4.2. Biological Image Processing

As a second application of ToloMEo, we discuss the case of biological images, where the progression of microscopy and multiplexed fluorescence imaging techniques allows one to take snapshots with enough resolution to distinguish cell populations [31,32,33,34] or even cellular compounds [35,36] and their respective spatial organization [37].

**Figure 4:**
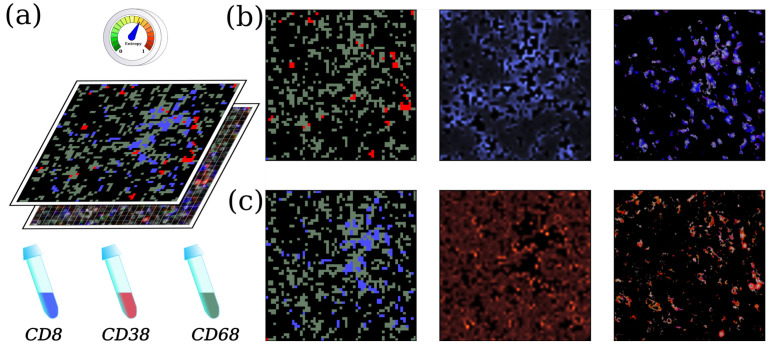
Biological imaging analysis. (**a**) Starting from a snapshot of the cell population taken from [31], four states are identified: environment (black), CD8-labeled cells (blue), CD38-positive cells (red), CD68-marked cells (green) and the ToloMEo method can be trained. (**b**) From left to right: (i) snapshot of the cell population without blue cells. (ii) Spatial probability distribution of finding a blue-labeled cell and (iii) spatial distribution of blue-labeled cells in the real image. (**c**) Same as in (**b**) but considering red-labeled cells in place of blue ones.

We applied ToloMEo considering the case of different cellular populations, where cells are labeled with different fluorescent markers. In particular, we started from an image proposed in Chevrier et al. [31], who, using fluorescent imaging on a tumor section, identified different types of macrophages and T cells present in the microenvironment of kidney cancer samples. We report the results in Figure 4.

Sampled tissues were stained with several fluorescence markers; in particular, the published image showed fluorescent signals for CD68 (green), CD38 (red), and CD8 (blue). The authors state that the samples used for imaging were highly enriched for macrophage and T cell phenotypes. Thus, cells expressing CD68 and CD8 fluorescences markers likely correspond to macrophage and T cells, respectively. On the other hand, CD38 (red marker) was co-expressed on both CD68 and CD8 cells, and these cells could co-localize.

To apply our method to the proposed image, we first segmented the picture, creating a grid of 57 × 57 cells. Then, we assigned each grid cell to one of four possible states according to the average color of the image pixels lying inside the grid cell. Black cells were considered the tumor microenvironment; green cells were considered macrophage cells, blue ones correspond to T cells, and red ones were considered control cells (see Figure 4a). While we expected a biologically relevant interaction between green and blue cells, red ones should be less correlated as they can be either macrophages or T cells. Once the grid states were properly assigned, we ran ToloMEo, with four states and the *‘lattice’* topology.

The final Hamiltonian we obtain is (states are ordered as black, green, red, blue):

(16) $$h=\left(\begin{array}{cccc} 0.64 & 1.54 & 4.30 & 2.23 \end{array}\right)\;,\;J=\left(\begin{array}{cccc} -0.112 & 0.320 & -0.036 & 0.532\\ 0.320 & -0.442 & 0.318 & 0.247\\ -0.036 & 0.318 & 0.047 & 0.060\\ 0.532 & 0.247 & 0.060 & -0.927 \end{array}\right)\;.$$

Interpreting the values of h and J directly is dangerous, since they have the gauge freedom we discussed in Section 3. However, comparing the relative values of the h and J, we still can extract useful information.

For example, red cells do not interact with most of the other cells, as represented by the third column of J, where the values for the red interactions are one order of magnitude smaller than the others. The only exception is the interaction between red and green cells, which is positive (repulsion), indicating that red and green cells prefer not to stay close. On the other side, we have the blue cells (last column), which interact the most with themselves (tend to form clusters). When inside the microtumoral environment (first column, black), the blue cells prefer to stay close to green cells as $J_{41}>J_{42}$.

As we explained in Section 3, ToloMEo can be applied to infer the position of the cells when information is missing. To show this feature of the program, in Figure 4b, we removed the blue cells from the image. Then, we run the Metropolis algorithm with the final Hamiltonian (*H*) found by ToloMEo, fixing the red and green cells and only simulating blue and black states. The software extracts $N_{\mathrm{conf}}$ configurations, and we predict the probability of finding the blue cells in each position. The comparison with the correct location of the blue cells is very good, confirming that ToloMEo can be actively employed to predict the system’s status. On the other hand, in Figure 4c, we remove the red cells, which are less correlated with the other cells. The prediction of the ToloMEo algorithm for the most likely location of red cells is much more uniform in the space, in qualitative accordance to the biological interpretation of the data (red cells can be either T cells or macrophages).

After the training, ToloMEo provides the entropy without any additional Metropolis calculation. The final entropy is S=0.54ln4. The mean-field $S_{\mathrm{SF}}$ contribution to the entropy is 0.58ln4. Thus, the correlations between species reduces the entropy by a 7%. This is a measure of the predictability of the system, where S=ln4 means complete randomness, while if S=0, ToloMEo performs a perfect prediction.

### 4.3. General Network Models: The Hopfield Neural Network

Finally, we apply our method to study the Hopfield model, a deterministic recurrent neural network (RNN) that describes the dynamics of a set of binary neurons [38,39]. In particular, we consider a network of *N* (=20) binary neurons interacting via a connectivity matrix *J*, with matrix elements $J_{ij}$ for i,j=1,…,N. The matrix element $J_{ij}$ represents the strength of the connection between the pre-synaptic neuron *j* and the post-synaptic neuron *i*. The state of each neuron is represented by a binary state variable, $\sigma_{i}$, that takes values either −1 or 1 if the neuron is, respectively, at rest (inactive) or firing (active). At each timestep, all neurons are updated synchronously [23,40] according to the discrete-time RNN evolution rule:

(17) $$\sigma_{i}\left(t+1\right)=\theta \left(\sum_{j}^{N}J_{ij}\sigma_{j}\left(t\right)-\eta_{i}\right),$$

where

(18) $$\theta \left(t\right)=\left\{\begin{array}{cc} -1 & \mathrm{if}\;t<0\\ U([-1,1]) & \mathrm{if}\;t=0\\ 1 & \mathrm{if}\;t>0 \end{array}\right.\;,$$

with U([−1,1]) meaning that when t=0, the function assumes value −1 or 1 with uniform distribution, while $\eta_{i}$ is a certain firing threshold. At the next step t+1, the neuron *i* fires (i.e., $\sigma_{i}\left(t+1\right)=1$), if the summation of its synaptic inputs is above the threshold $\eta_{i}$; otherwise, the neuron is inactive (i.e., $\sigma_{i}\left(t+1\right)=-1$). Here, we set $\eta_{i}=0$ for all neurons. The vector $\sigma \left(t\right)=(\sigma_{1}\left(t\right),\sigma_{2}\left(t\right),\ldots ,\sigma_{N}\left(t\right))$ represents the activation profile of all neurons at time *t*.

**Figure 5:**
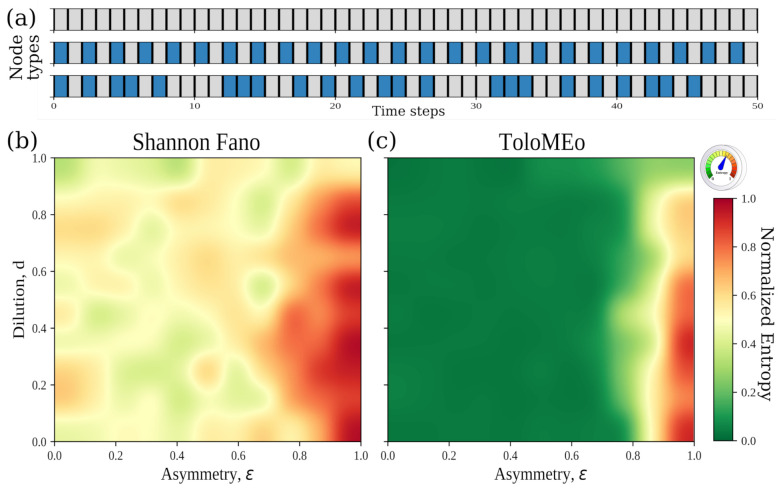
Entropy of the Hopfield neural network. (**a**) Examples of possible single neuron activation profiles. From top to bottom: always active neuron, oscillating neuron with period one, chaotic neuron. (**b**) Mean MaxEnt normalised entropy obtained as a function of the network asymmetry, ϵ and ailution, *d* for a Hopfield network of 20 nodes obtained in the Shannon–Fano approximation. Averages are performed over 10 independent realizations of the Hopfield dynamics for each couple of dilution and asymmetry parameters. (**c**) Same as in (**b**) but using the ToloMEo algorithm.

Finally, $J_{ij}$ quantifies the strength of the connection between neuron *i* and *j*. Following Folli et al. [23], we generate random connectivity matrices, *J*, as a function of two crucial network features, i.e., the level of network dilution *d* and coupling asymmetry ϵ. The network dilution measures the fraction of connected neuron couples, while the network asymmetry quantifies to what extent the underlying connectivity matrix is asymmetric. Operatively, we build the connectivity matrix, *J*, as:

(19) $$J=\left(1-\frac{\epsilon }{2}\right)S+\frac{\epsilon }{2}A,$$

where *S* (resp. *A*) is a symmetric (resp. asymmetric) matrix, whose off-diagonal elements are randomly sampled from a uniform distribution in the interval [−1,+1], while the diagonal elements are set to zeros (i.e., no autapse are present in the network [24]). The ϵ parameter can assume values in the interval [0,+2], measuring to what extent the underlying connectivity matrix is asymmetric. For ϵ=0, only the symmetric term of the *J* matrix remains, and thus neurons interact symmetrically with each other; if ϵ=2, *J* is fully asymmetric. Here, we explore the interval [0,1] along the lines of Folli et al. [23], i.e., we range from symmetrical to moderately asymmetrical networks. To account for network dilution, elements of the *J* matrix are set to zero, with probability *d* in such a way that the average number of links in a network with dilution *d* is $N_{d}=d\frac{N(N-1)}{2}$. Figure 5a shows three kinds of realizations of the Hopfield dynamics of single neurons, which start from random initial activation profiles ($\vec{\sigma }\left(t=0\right)$) and different connectivity matrices, *J*. As one can see, there can be nodes that remain active (or inactive) during the whole dynamics, can oscillate from active to inactive with a fixed period, or can give rise to chaotic dynamics.

To compactly assess the mean behavior of the network in different regimes of dilution and asymmetry, we generate random connectivity matrices varying both the dilution and asymmetry parameters. Then, we apply ToloMEo over the obtained single-neuron trajectories and estimate the mean entropies (Equation (15)) both in the Shannon–Fano and MaxEnt approximations (see Figure 5b,c). As one can see, both entropy maps show an increase in the entropy as the network becomes more asymmetric (ϵ→1) and fully connected (d→0). In particular, if we look at low dilutions and move along the asymmetry direction, the MaxEnt entropy exhibits an abrupt change, passing from an ordered region (low entropy) to a highly disordered one (high entropy) for ϵ∼0.8. This region is characterized by chaotic neuron dynamics [41] and, indeed, recurrent neural networks in the fully connected and fully asymmetric region exhibit a very low storage capacity with large basins of attraction, indicating the incapability of the network to distinguish different external stimuli [23]. It is worth noticing that, from a biological point of view, when an RNN drifts out of its optimal state from external causes such as the insurgence of a disease, the network becomes less effective in separating different stimuli and discriminating errors from signals. In line with these observations, it has been reported that the brain of patients affected by autism spectrum disorders presents an altered dilution compared to healthy individuals [42].

On the other hand, asymmetric and diluted connectivity matrices exhibit optimal storage capacity, meaning that a significant fraction of elements in the connectivity matrix are zero. Such connectivity features are observed in biological cases, such as in the neocortex and hippocampus regions, and are implicated in memory storage and retrial [43,44,45].

Here, we showed that the dynamical entropy computed with ToloMEo correctly describes the quantity of information a recurrent neural network can store.

## 5. Conclusions

We presented ToloMEo, a novel algorithm able to infer the maximum entropy probability distribution of the discrete states of a network. The method can be applied to a wide variety of systems. We revised its application in ecosystem dynamics and presented for the first time its application in image processing. Indeed, we employed ToloMEo to infer the effective interaction between macrophages and T cells in kidney cancer samples and showed how to infer the most likely arrangement of cells in the absence of markers. Then, we employed the dynamic extension of maximum entropy to study the complexity transition in the trajectory of the Hopfield model. The method efficiently models even out-of-equilibrium processes and transient dynamics, enabling the characterization of the transition between chaotic trajectory and attractors. Furthermore, we showed how the dynamical entropy computed with ToloMEo correctly indicates the quantity of information a recurrent neural network can store, paving the way for the systematic employment of the method to assess the quality of the network.

The ToloMEo method is released as a web server app, freely accessible at http://circe.iit.uniroma1.it:9205/ (25 August 2021).

## Data Availability

The data employed in this work are available from the authors upon reasonable request.

## Abbreviations

The following abbreviations are used in this manuscript:

SF	Shannon–Fano
MaxEnt	Maximum Entropy
ToloMEo	TOpoLogical netwOrk Maximum Entropy Optimiziation
NESS	Non-Equilibrium Steady-State
RNN	Recurrent Neural Networks
